# Supplementary material for: End Sequence Analysis Toolkit (ESAT) expands the extractable information from single-cell RNA-seq data
Source: Genome Res. 2016 Oct;26(10):1397–410. doi: 10.1101/gr.207902.116 (PMC5052061; doi:10.1101/gr.207902.116)
Supplement: Supplemental Material [file supp_gr.207902.116_Supp_Materials.tar.gz › ESAT-esat_v0.1/doc/html/classbroad_1_1core_1_1math_1_1_empirical_distribution-members.html]

ESAT: Member List


ESAT


- Main Page
- Related Pages
- Packages
- Classes
- Files

- Class List
- Class Index
- Class Hierarchy
- Class Members

All Classes Namespaces Files Functions Variables Enumerator Pages

- broad
- core
- math
- EmpiricalDistribution

broad.core.math.EmpiricalDistribution Member List

This is the complete list of members for broad.core.math.EmpiricalDistribution, including all inherited members.

|  |  |  |
| --- | --- | --- |
| add(double observation) | broad.core.math.EmpiricalDistribution |  |
| addAll(Collection< Double > values) | broad.core.math.EmpiricalDistribution |  |
| addAll(double[] values) | broad.core.math.EmpiricalDistribution |  |
| addAll(Matrix vals) | broad.core.math.EmpiricalDistribution |  |
| addDistribution(EmpiricalDistribution other) | broad.core.math.EmpiricalDistribution |  |
| addPsuedocounts(double psuedocount) | broad.core.math.EmpiricalDistribution |  |
| chiSquareDistance(EmpiricalDistribution other) | broad.core.math.EmpiricalDistribution |  |
| EmpiricalDistribution(int numberOfBins, double min, double max) | broad.core.math.EmpiricalDistribution |  |
| EmpiricalDistribution(int numberOfBins, double min, double max, boolean includeOutOfRange) | broad.core.math.EmpiricalDistribution |  |
| EmpiricalDistribution(Collection< Double > vals, int numberOfBins) | broad.core.math.EmpiricalDistribution |  |
| EmpiricalDistribution(Collection< Double > vals) | broad.core.math.EmpiricalDistribution |  |
| EmpiricalDistribution(Collection< Double > vals, int numberOfBins, boolean absVal) | broad.core.math.EmpiricalDistribution |  |
| EmpiricalDistribution(double[] vals, int numberOfBins) | broad.core.math.EmpiricalDistribution |  |
| EmpiricalDistribution(List< Double > vals, int numberOfBins) | broad.core.math.EmpiricalDistribution |  |
| EmpiricalDistribution(double[] vals, int numberOfBins, double min, double max) | broad.core.math.EmpiricalDistribution |  |
| EmpiricalDistribution(InputStream is) | broad.core.math.EmpiricalDistribution |  |
| EmpiricalDistribution(File source) | broad.core.math.EmpiricalDistribution |  |
| EmpiricalDistribution(Matrix permutations, int numberOfBins) | broad.core.math.EmpiricalDistribution |  |
| ensureNoEmptyBins() | broad.core.math.EmpiricalDistribution |  |
| getAllDataValues() | broad.core.math.EmpiricalDistribution |  |
| getBin(double value) | broad.core.math.EmpiricalDistribution |  |
| getBinEnd(int bin) | broad.core.math.EmpiricalDistribution |  |
| getBinMidPoint(int bin) | broad.core.math.EmpiricalDistribution |  |
| getBinNumber() | broad.core.math.EmpiricalDistribution |  |
| getBinStart(int bin) | broad.core.math.EmpiricalDistribution |  |
| getCountByBinStart() | broad.core.math.EmpiricalDistribution |  |
| getCumulativeProbability(double value) | broad.core.math.EmpiricalDistribution |  |
| getDensity(int bin) | broad.core.math.EmpiricalDistribution |  |
| getDistributionOfHistogramValues() | broad.core.math.EmpiricalDistribution |  |
| getEmpiricalMaxStatisticDistribution(Collection< EmpiricalDistribution > distributions) | broad.core.math.EmpiricalDistribution | static |
| getFirstIntersectLeftFromMedian(EmpiricalDistribution other) | broad.core.math.EmpiricalDistribution |  |
| getFirstIntersectRightFromMedian(EmpiricalDistribution other) | broad.core.math.EmpiricalDistribution |  |
| getHistogram(int bin) | broad.core.math.EmpiricalDistribution |  |
| getKSStatistic(List< Double > observed) | broad.core.math.EmpiricalDistribution |  |
| getKSStatistic(double[] observed) | broad.core.math.EmpiricalDistribution |  |
| getMax() | broad.core.math.EmpiricalDistribution |  |
| getMean() | broad.core.math.EmpiricalDistribution |  |
| getMedianOfAllDataValues() | broad.core.math.EmpiricalDistribution |  |
| getMin() | broad.core.math.EmpiricalDistribution |  |
| getNumberOfValuesLargerThan(double value) | broad.core.math.EmpiricalDistribution |  |
| getPercentOfValuesBetween(double leftBound, double rightBound) | broad.core.math.EmpiricalDistribution |  |
| getPercentOfValuesLargerThan(double value) | broad.core.math.EmpiricalDistribution |  |
| getProbability(double size) | broad.core.math.EmpiricalDistribution |  |
| getPValue(double score) | broad.core.math.EmpiricalDistribution |  |
| getPValue(double score, double min) | broad.core.math.EmpiricalDistribution |  |
| getPValue2(double score) | broad.core.math.EmpiricalDistribution |  |
| getQuantileOfBins(double probability) | broad.core.math.EmpiricalDistribution |  |
| getRawDensity(int bin) | broad.core.math.EmpiricalDistribution |  |
| getStandardDeviation() | broad.core.math.EmpiricalDistribution |  |
| getTotalObservations() | broad.core.math.EmpiricalDistribution |  |
| getZscore(double value) | broad.core.math.EmpiricalDistribution |  |
| KLDivergence(EmpiricalDistribution other) | broad.core.math.EmpiricalDistribution |  |
| KLDivergenceSym(EmpiricalDistribution other) | broad.core.math.EmpiricalDistribution |  |
| leftIntersect(EmpiricalDistribution other) | broad.core.math.EmpiricalDistribution |  |
| main(String[] args) | broad.core.math.EmpiricalDistribution | static |
| numBins() | broad.core.math.EmpiricalDistribution |  |
| rightIntersect(EmpiricalDistribution other) | broad.core.math.EmpiricalDistribution |  |
| testGoodnessOfFit(EmpiricalDistribution other) | broad.core.math.EmpiricalDistribution |  |
| USAGE | broad.core.math.EmpiricalDistribution | static |
| write(BufferedWriter bw) | broad.core.math.EmpiricalDistribution |  |
| write(String save) | broad.core.math.EmpiricalDistribution |  |


---

Generated on Fri Aug 15 2014 15:10:52 for ESAT by  

 1.8.7
